# Epstein–Barr Virus‐Positive B‐Cell Lymphoproliferative Disorder Complicated by Septic Shock in Activated PI3Kδ Syndrome: A Pediatric Case Report and Literature Review

**DOI:** 10.1155/crh/3644513

**Published:** 2026-08-11

**Authors:** Xumeng Zhao, Xi Ming, Zhen Shang, Mi Zhou, Yi Xiao

**Affiliations:** ^1^ Department of Hematology, Tongji Hospital, Tongji Medical College, Huazhong University of Science and Technology, Wuhan, Hubei, China, hust.edu.cn

**Keywords:** activated phosphoinositide 3-kinase δ syndrome, B-cell lymphoproliferative disorder, case report, Epstein–Barr virus, septic shock

## Abstract

Activated phosphoinositide 3‐kinase delta syndrome (APDS) is a rare inborn error of immunity caused by gain‐of‐function variants in *PIK3CD* and characterized by recurrent infections, lymphoproliferation, and impaired viral control. We report a 17‐year‐old male with a heterozygous *PIK3CD* c.3061G > A (p.E1021K) variant who presented with progressive edema, extensive hypermetabolic lymphadenopathy, splenomegaly, and Epstein–Barr virus (EBV) DNAemia. A core needle biopsy of the right inguinal lymph node demonstrated an immunodeficiency‐associated EBV‐positive B‐cell lymphoproliferative disorder with extensive monotypic plasmacytoid differentiation. Because the biopsy contained limited mature B‐cell tissue, the pathological findings favored a polymorphic B‐LPD although EBV‐positive diffuse large B‐cell lymphoma with plasmacytic differentiation could not be excluded. The patient received anti‐B‐cell‐directed therapy and supportive treatment. He was subsequently readmitted with septic shock and acute respiratory distress syndrome. Blood metagenomic next‐generation sequencing detected *Escherichia coli* and *Klebsiella pneumoniae*, together with antimicrobial‐resistance genes including *blaNDM*. Despite intensive antimicrobial and organ‐supportive treatment, the patient remained critically ill and was discharged at his family’s request for transfer to a local hospital; his subsequent outcome was unavailable. This case highlights the diagnostic difficulty of classifying EBV‐positive B‐cell proliferations using limited biopsy tissue in APDS and the competing risks of lymphoproliferative disease and severe infection. Adequate tissue sampling and pathological characterization, close microbiological surveillance, and individualized multidisciplinary management are essential in this setting.

## 1. Introduction

Activated phosphoinositide 3‐kinase δ syndrome (APDS) is a rare inborn error of immunity caused by germline gain‐of‐function mutations in *PIK3CD*, typically inherited in an autosomal dominant manner [1, 2]. PI3Kδ is predominantly expressed in hematopoietic cells, and persistent activation of this pathway disrupts T‐ and B‐cell differentiation and immune homeostasis, resulting in recurrent infections, lymphoproliferation, and immune dysregulation [1–3]. Children with APDS commonly present with recurrent respiratory tract infections and impaired Epstein–Barr virus (EBV) control, while growth retardation and developmental delay may also occur [1–4]. Chronic immune dysregulation and defective viral control confer an increased risk of EBV‐associated lymphoproliferative disorders and B‐cell lymphomas [2, 3]. Although this association is increasingly recognized, evidence regarding the pathological classification, treatment, and outcomes of APDS‐associated EBV‐positive B‐cell lymphoproliferative disorders (B‐LPDs) remains limited, and no standardized management strategy has been established.

This report describes a pediatric patient with APDS who developed an immunodeficiency‐associated EBV‐positive B‐LPD complicated by severe infection. By presenting the clinical course, diagnostic evaluation, and therapeutic management of this patient and reviewing selected previously reported cases, we examine EBV‐associated lymphoproliferation within the broader clinical spectrum of APDS (Table 1). This report aims to improve recognition of this uncommon complication and inform individualized diagnostic and therapeutic decision‐making.

**TABLE 1:** Summary of reported cases of activated PI3Kδ syndrome.

Report	Sex	Age	PIK3CD variant	Clinical manifestations	Treatment	Outcome
This case	Male	17 y	c.3061G > A (p.E1021K)	Recurrent infections, growth retardation, generalized lymphadenopathy, splenomegaly, and EBV‐positive immunodeficiency‐associated B‐LPD (polymorphic subtype favored; DLBCL not excluded)	Ripertamab, rituximab, and polatuzumab vedotin; zanubrutinib prescribed at discharge; antimicrobial and organ‐supportive therapy	Septic shock and acute respiratory distress syndrome; alive at transfer, with subsequent outcome unavailable
Jiang et al. [5]	Male	1 y 5 mo	c.3061G > A (p.E1021K)	Fever, rash, alopecia, recurrent respiratory infections, hepatosplenomegaly, lymphadenopathy, vomiting, diarrhea, anemia, thrombocytopenia, low IgG and IgA, elevated IgM, reduced CD19, focal cutaneous depigmentation	Sirolimus	Marked improvement of hepatosplenomegaly
Jiang et al. [5]	Female	—	c.2314G > A	SLE‐like manifestations, refractory thrombocytopenia	Sirolimus	Improvement of thrombocytopenia
Craig et al. [6]	Male	9 y	c.3061G > A (p.E1021K)	Growth retardation with growth hormone deficiency, recurrent sinopulmonary infections, bronchiectasis, wheezing, lymphadenopathy, splenomegaly, impaired specific antibody responses	Growth hormone replacement, immunoglobulin replacement, antibiotic therapy, bronchodilator therapy	Stable
Zhang et al. [7]	Male	4 y	c.1574A > G (p.E525G)	Recurrent pneumonia, hematuria, hepatosplenomegaly, sinusitis, lymphadenopathy, bronchial mucosal cobblestone‐like changes, EBV infection, ANCA positivity	Glucocorticoids, cyclophosphamide, mycophenolate mofetil, anti‐infective therapy, intravenous immunoglobulin	Marked reduction of lymphadenopathy and hepatosplenomegaly, with improvement of hematuria
Cansever et al. [8]	Male	2.5 y	c.3061G > A (p.E1021K)	Fever, skin lesions, hepatosplenomegaly, lymphadenopathy, thrombocytopenia, hemolytic anemia, HLH, recurrent respiratory infections, CMV/EBV infections, Hodgkin lymphoma	Chemotherapy (cyclophosphamide, vincristine, etoposide, rituximab), allo‐HSCT	Successful transplantation, good outcome at 18‐month follow‐up
Luo et al. [9]	Female	6 y	c.1570T > A (p.Y524N)	Recurrent upper respiratory infections, bronchiectasis, lymphoproliferation, EBV infection, mucosal lymphoid hyperplasia	Sirolimus	Improvement of lymphoproliferation
Pham and Cunningham‐Rundles [10]	Female	2 y	c.3061G > A (p.E1021K)	Recurrent pneumonia, bronchiectasis, low IgG and IgA, elevated IgM, T/B cell lymphopenia, lymphadenopathy, splenomegaly, anemia, EBV infection	Anti‐infective therapy, immunoglobulin replacement, rituximab, sirolimus	Reduction of lymphadenopathy (symptoms recurred after treatment interruption)
Hong et al. [11]	Male	13 y	c.3061G > A (p.E1021K)	High fever, hepatosplenomegaly, lymphadenopathy, anemia, thrombocytopenia, intestinal lymphoid hyperplasia causing recurrent intussusception, recurrent respiratory infections, bronchitis, CMV and EBV infections	allo‐HSCT, immunosuppressive therapy, anti‐infective therapy	Successful transplantation, good outcome at 2‐year follow‐up
Bucciol et al. [12]	Female	15 y	c.3061G > A (p.E1021K)	Recurrent respiratory infections, bronchiectasis, hypogammaglobulinemia, T‐cell lymphopenia and senescence, lymphadenopathy, EBV infection, papillary thyroid carcinoma	Anti‐infective therapy, immunoglobulin replacement, bronchiectasis management, total thyroidectomy with lymph node dissection, radiotherapy	Stable, no relapse

*Note:* APDS, activated PI3Kδ syndrome; allo‐HSCT, allogeneic hematopoietic stem cell transplantation; CMV, cytomegalovirus; HLH, hemophagocytic lymphohistiocytosis; Ig, immunoglobulin.

Abbreviations: DLBCL, diffuse large B‐cell lymphoma; EBV, Epstein–Barr virus; SLE, systemic lupus erythematosus.

## 2. Case Presentation

**FIGURE 1:**
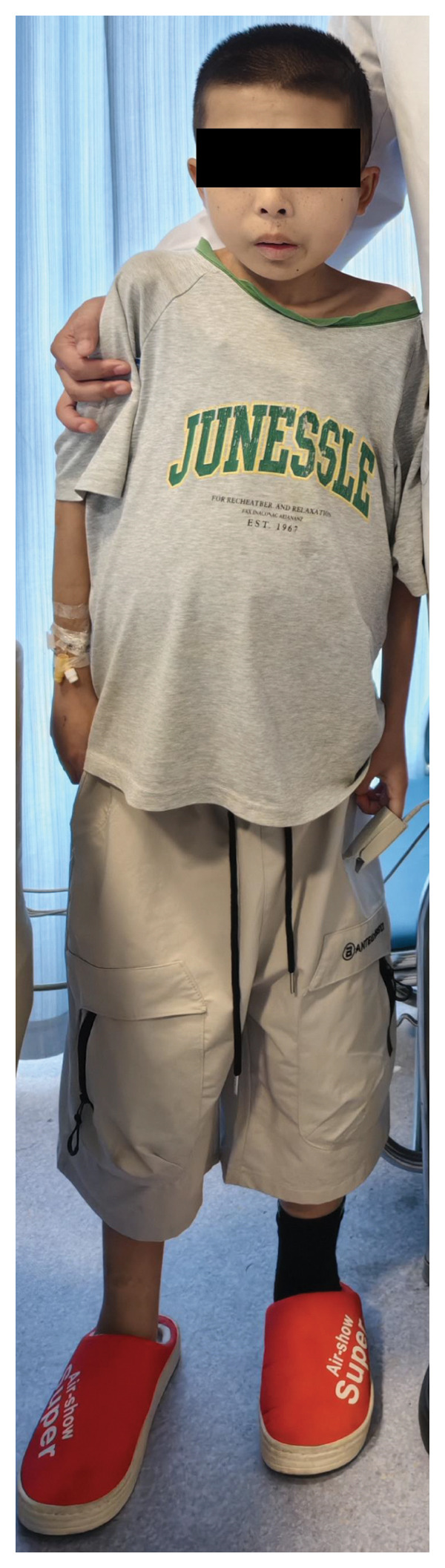
Clinical appearance demonstrating short stature in the patient with APDS.

A 17‐year‐old Chinese male had been diagnosed with a *PIK3CD*‐associated inborn error of immunity in 2013 and subsequently received long‐term oral glucocorticoid therapy. Intermittent intravenous immunoglobulin therapy had been administered since 2017. At the current presentation, his height was 140 cm and body weight was 32 kg, both markedly below the age‐ and sex‐specific reference ranges and consistent with growth retardation (Figure 1). Cognitive impairment was also clinically noted. Both parents were reportedly unaffected, and there was no known consanguinity or family history of a similar disorder. The patient had documented allergies to piperacillin and azithromycin.

On June 20, 2025, the patient developed scrotal pain without an identifiable trigger and was evaluated at a local hospital, where no specific intervention was initiated. Approximately 2 weeks later, the pain worsened. Laboratory testing showed a hemoglobin concentration of approximately 60 g/L, and abdominal ultrasonography revealed enlarged splenic‐hilar lymph nodes. Referral to a tertiary hospital was recommended, but no PET‐CT or biopsy was performed at that time. Scrotal swelling subsequently progressed, followed by facial and left lower‐extremity edema.

On September 20, 2025, the patient developed difficulty urinating in the setting of marked scrotal and generalized edema. The urinary difficulty and edema improved transiently after albumin infusion and diuretic therapy at a local hospital but worsened again after discharge. On September 27, 2025, he was admitted to our Department of Hematology because of persistent scrotal pain for approximately 3 months and progressive edema.

On admission, physical examination revealed marked short stature and edema involving the face, scrotum, and bilateral lower extremities. Laboratory testing showed anemia (hemoglobin, 84 g/L), hypoalbuminemia (30.0 g/L), an elevated high‐sensitivity C‐reactive protein level (106.7 mg/L), electrolyte abnormalities, and coagulation abnormalities. Samples collected on September 27 showed EBV‐DNA loads of 1.26 × 10^4^ copies/mL in peripheral blood mononuclear cells and 1.48 × 10^4^ copies/mL in plasma.

Bone marrow cytology showed trilineage hyperplasia without abnormal lymphoid proliferation. Flow cytometric immunophenotyping detected no clonal B‐cell or plasma‐cell population, providing no evidence of bone marrow involvement by the B‐LPD.

PET‐CT performed on September 30 demonstrated extensive hypermetabolic lymphadenopathy involving the cervical, supraclavicular, mediastinal, hilar, abdominal, retroperitoneal, pelvic, and inguinal regions, accompanied by splenomegaly with increased metabolic activity. Wall thickening involving multiple colonic segments with increased metabolic uptake, bilateral pleural effusions, ascites, and diffuse subcutaneous edema were also observed. These findings were consistent with systemic lymphoproliferative disease.

An ultrasound‐guided core needle biopsy of the right inguinal lymph node was performed on October 9, 2025. Histopathological and immunohistochemical examination demonstrated an immunodeficiency‐associated EBV‐positive B‐LPD with extensive monotypic plasmacytoid differentiation. The cells expressed CD19, CD79a, and CD79b, with diffuse expression of MUM1 and CD38. CD138 was partially expressed, whereas CD20 was detected in only a small subset. Lambda light‐chain restriction was demonstrated by positivity for lambda and negativity for kappa. EBER chromogenic in situ hybridization was positive, and the Ki‐67 proliferation index was approximately 90%. BCL6, CD10, BCL2, and EBNA2 were negative, whereas MYC was expressed in approximately 10% of cells. Tissue flow cytometry identified an abnormal lambda light‐chain–restricted mature B‐cell population accounting for approximately 45% of the analyzed nucleated cells and expressing CD45, CD19, CD20, CD79b, and CD38. Because the core biopsy contained limited mature B‐cell tissue, the pathological findings favored a polymorphic B‐LPD, although EBV‐positive diffuse large B‐cell lymphoma (DLBCL) with plasmacytic differentiation could not be excluded. An excisional biopsy was recommended if clinically feasible. Given the limited core‐biopsy tissue and the inability to establish a definitive diagnosis of DLBCL, Hans cell‐of‐origin classification was not applied. Fluorescence in situ hybridization for *MYC, BCL2*, and *BCL6* rearrangements was not performed.

Whole‐exome sequencing of peripheral blood during the current admission detected a heterozygous *PIK3CD* variant, NM_005026.5:c.3061G > A (p.E1021K), at a variant allele frequency of 51.5%. The variant was interpreted as germline in the context of the patient’s established APDS phenotype although it was not independently confirmed in a nonhematopoietic sample. Paired tumor–normal sequencing was not performed. Collectively, the clinical, pathological, and molecular findings supported APDS complicated by an EBV‐positive immunodeficiency‐associated B‐LPD.

Given the extensive disease burden and the patient’s compromised clinical condition, anti‐B‐cell‐directed treatment was initiated during the diagnostic evaluation. Ripertamab, an anti‐CD20 monoclonal antibody, was administered on October 6, 7, and 10 at a cumulative dose of 250 mg; rituximab was administered on October 8 and 9 at a cumulative dose of 200 mg; and polatuzumab vedotin was administered at 30 mg on October 9. Zanubrutinib was prescribed at discharge. No CHOP‐ or EPOCH‐based cytotoxic regimen was administered. Supportive treatment included organ‐protective measures, hematopoietic growth‐factor support, and anti‐infective therapy. At discharge on October 11, the prescribed medications included trimethoprim–sulfamethoxazole, contezolid, voriconazole, cefdinir, and methylprednisolone. Repeat EBV testing on October 11 detected 1.449 × 10^4^ copies/mL in whole blood and 1.247 × 10^3^ copies/mL in plasma. Cell‐subset analysis detected 4.680 × 10^3^ EBV copies per 2 × 10^5^ peripheral blood mononuclear cells and 2.099 × 10^5^ copies per 2 × 10^5^ B cells, whereas EBV DNA was undetectable in the tested T‐cell and natural killer‐cell fractions.

Fever, cough, and abdominal distension developed on October 14, and the patient was readmitted to our hospital on October 17 with septic shock and acute respiratory distress syndrome. He was not neutropenic at readmission (absolute neutrophil count, 8.47 × 10^9^/L). Abdominal distension occurred in the setting of known ascites, splenomegaly, and generalized edema. No repeat PET‐CT or other response‐assessment imaging was performed; therefore, the response of the B‐LPD to treatment could not be evaluated. Blood metagenomic next‐generation sequencing detected *Escherichia coli* (260,132 reads; relative abundance, 53.1%) and *Klebsiella pneumoniae* (1449 reads; relative abundance, 0.9%), together with the resistance genes *blaCTX-M, blaNDM*, and *blaSHV*. Screening for carbapenem‐resistant *E. coli* was positive, and a stool assay detected the *Clostridioides difficile* toxin B gene. *Pneumocystis jirovecii* DNA and respiratory viral testing were negative, and fungal culture of a suboptimal sputum specimen was negative. Although no respiratory pathogen was definitively identified, the clinical and imaging findings supported a severe pulmonary infection. Antimicrobial treatment was adjusted to ceftazidime–avibactam plus aztreonam and amphotericin B. Additional treatment included fluid resuscitation, vasopressor support, continuous renal replacement therapy, albumin supplementation, hepatic support, and nutritional support. Despite partial improvement in inflammatory markers, the patient remained critically ill. At the family’s request, he was discharged against medical advice on October 27 for transfer to a local hospital. He was alive at transfer, but information regarding subsequent treatment and outcome was unavailable.

## 3. Discussion

APDS is a rare inborn error of immunity caused by germline gain‐of‐function variants in *PIK3CD* and is most commonly inherited in an autosomal dominant manner [2, 3]. The p.E1021K variant identified in this patient is the most extensively characterized pathogenic hotspot and is strongly associated with the classical APDS phenotype [3]. Constitutive activation of PI3Kδ leads to persistent dysregulation of the PI3K/AKT/mTOR signaling pathway, which has a central role in lymphocyte development and immune homeostasis [3]. Because PI3Kδ is predominantly expressed in hematopoietic cells, sustained pathway activation disrupts B‐cell maturation and T‐cell differentiation, thereby producing the combined immune deficiency and immune dysregulation characteristic of APDS.

The increasing availability of high‐throughput sequencing has facilitated recognition of APDS as a complex immune disorder characterized by recurrent infections, lymphoproliferation, and impaired viral control [1, 4, 13]. Recurrent upper and lower respiratory tract infections are among the earliest and most common manifestations and often begin in infancy. Their development reflects a combination of impaired humoral immunity, premature T‐cell senescence, and compromised mucosal immune defense associated with sustained PI3Kδ activation [13, 14]. Impaired EBV control is another prominent feature and has been documented in multinational APDS cohorts [4, 15, 16]. This abnormality does not necessarily result from a lack of EBV‐specific CD8^+^ T cells, which may be preserved or increased, but may instead reflect defective effector differentiation and impaired maintenance of antiviral function [17]. In the present case, serial EBV‐DNA measurements during hospitalization and EBER positivity in the lymph‐node biopsy supported an association between impaired EBV control and the observed B‐cell proliferation. However, the absence of quantitative EBV measurements before the current clinical episode precluded determination of the duration of EBV DNAemia and its temporal relationship with the development of B‐LPD.

Lymphoproliferation represents another defining manifestation of APDS. Persistent PI3Kδ activation promotes B‐cell survival and proliferation, while chronic antigenic stimulation contributes to enlargement of peripheral and mucosa‐associated lymphoid tissues [17]. Over time, the resulting immune dysregulation and proproliferative microenvironment may facilitate malignant transformation. Epidemiological studies have identified APDS as an inborn error of immunity associated with a particularly high risk of lymphoma, especially EBV‐associated B‐cell lymphoma [2, 3, 18, 19]. In the present patient, the lymph‐node core biopsy demonstrated an EBV‐positive, monotypic B‐cell proliferation with extensive plasmacytoid differentiation. The pathological findings favored a polymorphic B‐LPD; however, the limited amount of mature B‐cell tissue precluded definitive exclusion of EBV‐positive DLBCL with plasmacytic differentiation. This diagnostic uncertainty illustrates the limitations of core needle biopsy in heterogeneous EBV‐associated lymphoid proliferations and supports obtaining an excisional biopsy whenever clinically feasible.

Growth impairment is an additional clinically relevant but sometimes underrecognized manifestation of APDS. Recurrent infection, chronic inflammation, sustained pathway dysregulation, nutritional compromise, and endocrine abnormalities may collectively contribute to impaired somatic development [1]. The marked short stature, low body weight, and clinically evident cognitive impairment in this patient further illustrate the multisystem nature of APDS and its consequences beyond recurrent infection and lymphoproliferation.

The management of EBV‐positive B‐LPD in APDS requires a careful balance between disease control and the risk of treatment‐related infection in the setting of pre‐existing immune dysfunction. Evidence guiding lymphoma‐directed treatment in APDS remains limited to case reports and small cohorts, and no standard regimen has been established. Anti‐CD20, anti‐CD79b, and BTK‐directed therapies may represent potential treatment options, but their efficacy and safety in APDS‐associated B‐LPD remain insufficiently defined. Experience from other immunodeficiency‐associated aggressive B‐cell lymphomas provides relevant insights. Short‐course EPOCH‐based regimens have achieved favorable outcomes in patients with HIV‐associated DLBCL and Burkitt lymphoma, suggesting that risk‐adapted immunochemotherapy may be feasible in selected immunodeficient patients [20, 21]. However, these findings cannot be directly extrapolated to APDS, particularly in patients with active infection, limited physiological reserve, or incompletely classified B‐LPD. In the present case, anti‐B‐cell‐directed therapy was selected in the setting of extensive disease burden and a compromised clinical condition. The subsequent development of severe infection despite antimicrobial prophylaxis highlights the difficulty of balancing disease control against infectious risk and underscores the importance of individualized, multidisciplinary treatment decisions.

Disease‐directed treatment of APDS has increasingly focused on suppressing aberrant PI3Kδ signaling. The mTOR inhibitor sirolimus can reduce non‐neoplastic lymphoproliferative manifestations, including lymphadenopathy and splenomegaly, although correction of the underlying immune dysfunction is often incomplete and persistent viral replication may continue [5, 22]. More recently, the selective PI3Kδ inhibitor leniolisib has been shown to attenuate mutation‐driven pathway hyperactivation, reduce lymphadenopathy and splenomegaly, and improve selected immunological abnormalities [2]. Nevertheless, its long‐term effects on lymphoma risk remain uncertain, and the roles of sirolimus and leniolisib in patients with established EBV‐associated B‐LPD have not been defined.

Allogeneic hematopoietic stem cell transplantation (allo‐HSCT) is currently the only treatment capable of replacing the defective immune system and providing definitive immune reconstitution in APDS [23]. It may be considered in patients with refractory or recurrent infections, progressive lymphoproliferation, severe immune dysregulation, or malignant transformation [11, 24, 25]. Available reports indicate that allo‐HSCT can provide durable immune correction, including in selected patients undergoing alternative‐donor transplantation [11, 24, 25]. However, transplantation carries substantial risks, and its timing requires careful consideration. Active infection, uncontrolled lymphoproliferative disease, poor nutritional status, and organ dysfunction may markedly increase transplant‐related morbidity and mortality. Earlier identification of high‐risk patients may, therefore, preserve the opportunity to consider allo‐HSCT before severe infection or irreversible organ damage develops.

This case illustrates the diagnostic uncertainty associated with EBV‐positive B‐cell proliferations evaluated using limited core‐biopsy tissue and the competing risks of lymphoproliferative disease and uncontrolled infection in APDS. Interpretation is limited by the absence of longitudinal EBV monitoring before the current clinical episode, the limited lymph‐node tissue available for definitive classification, the lack of formal treatment–response assessment, and unavailable follow‐up after transfer. Nevertheless, the case emphasizes the importance of early genetic evaluation in patients with compatible immune phenotypes, longitudinal EBV monitoring, acquisition of adequate tissue for pathological classification whenever clinically feasible, and close microbiological surveillance during treatment. Because evidence guiding B‐LPD‐directed therapy in APDS remains limited, treatment should be individualized through multidisciplinary assessment of pathological classification, disease burden, immune status, infectious risk, and eligibility for definitive immune reconstitution.

**Nomenclature**
APDS: Activated phosphoinositide 3‐kinase δ syndrome
ARDS: Acute respiratory distress syndrome
Allo‐HSCT: Allogeneic hematopoietic stem cell transplantation
B‐LPD: B‐cell lymphoproliferative disorder
DLBCL: Diffuse large B‐cell lymphoma
EBV: Epstein–Barr virus
PET‐CT: Positron emission tomography–computed tomography

## Author Contributions

Xumeng Zhao collected and interpreted the clinical data and drafted the manuscript. Xi Ming, Zhen Shang, Mi Zhou, and Yi Xiao contributed to clinical data interpretation and critically revised the manuscript. Yi Xiao supervised the work.

## Funding

This work was supported by the National Natural Science Foundation of China (nos. 81873444, 82070213, and 82370196 to Yi Xiao).

## Disclosure

All authors have read and approved the final version of the manuscript. Yi Xiao, the corresponding author and manuscript guarantor, had full access to all of the data in this study and takes complete responsibility for the integrity of the data and the accuracy of the data analysis.

## Ethics Statement

Ethical statement was not required for this single‐patient case report in accordance with institutional policy.

## Consent

Written informed consent for publication of the clinical information and potentially identifiable image was obtained from the patient’s legal guardian.

## Conflicts of Interest

The authors declare no conflicts of interest.

## Supporting Information

Additional supporting information can be found online in the Supporting Information section.

## Supporting information


**Supporting Information** CARE_Checklist.

## Data Availability

The deidentified data supporting this case report are available from the corresponding author upon reasonable request, subject to institutional and patient privacy requirements.
