# Supplementary material for: Epstein–Barr Virus‐Positive B‐Cell Lymphoproliferative Disorder Complicated by Septic Shock in Activated PI3Kδ Syndrome: A Pediatric Case Report and Literature Review
Source: Case Rep Hematol. 2026 Aug 11;2026:3644513. doi: 10.1155/crh/3644513 (PMC13462656; doi:10.1155/crh/3644513)
Supplement: Supplementary file 1 [file CRH-2026-3644513-s001.pdf]

CARE case report guidelines

CARE Checklist of information to include when writing a case report

(cc) BY-NC-ND

Topic	Item	Checklist item description	Reported on Line
**Title**	1	The diagnosis or intervention of primary focus followed by the words "case report"	Lines 1-3
**Key Words**	2	2 to 5 key words that identify diagnoses or interventions in this case report, including "case report"	Lines 31-32
**Abstract (no references)**	3a	Introduction: What is unique about this case and what does it add to the scientific literature?	Lines 10-12
	3b	Main symptoms and/or important clinical findings	Lines 12-19
	3c	The main diagnoses, therapeutic interventions, and outcomes	Lines 15-25
	3d	Conclusion—What is the main "take-away" lesson(s) from this case?	Lines 26-30
**Introduction**	4	One or two paragraphs summarizing why this case is unique (**may include references**)	Lines 33-53
**Patient Information**	5a	De-identified patient specific information.	Lines 55-62
	5b	Primary concerns and symptoms of the patient.	Lines 65-76
	5c	Medical, family, and psycho-social history including relevant genetic information	Lines 55-62, 109-115
	5d	Relevant past interventions with outcomes	Lines 56-57, 116-124
**Clinical Findings**	6	Describe significant physical examination (PE) and important clinical findings.	Lines 58-62, 77-80
**Timeline**	7	Historical and current information from this episode of care organized as a timeline	Lines 55-148
**Diagnostic Assessment**	8a	Diagnostic testing (such as PE, laboratory testing, imaging, surveys).	Lines 77-115, 125-141
	8b	Diagnostic challenges (such as access to testing, financial, or cultural)	Lines 92-108
	8c	Diagnosis (including other diagnoses considered)	Lines 92-115
	8d	Prognosis (such as staging in oncology) where applicable	N/A
**Therapeutic Intervention**	9a	Types of therapeutic intervention (such as pharmacologic, surgical, preventive, self-care)	Lines 116-124, 142-144
	9b	Administration of therapeutic intervention (such as dosage, strength, duration)	Lines 117-124, 142-144
	9c	Changes in therapeutic intervention (with rationale)	Lines 129-144
**Follow-up and Outcomes**	10a	Clinician and patient-assessed outcomes (if available)	Lines 129-148
	10b	Important follow-up diagnostic and other test results	Lines 125-141
	10c	Intervention adherence and tolerability (How was this assessed?)	N/A
	10d	Adverse and unanticipated events	Lines 129-148
**Discussion**	11a	A scientific discussion of the strengths AND limitations associated with this case report	Lines 149-240
	11b	Discussion of the relevant medical literature **with references**.	Lines 149-240
	11c	The scientific rationale for any conclusions (including assessment of possible causes)	Lines 149-240
	11d	The primary "take-away" lessons of this case report (without references) in a one paragraph conclusion	Lines 229-240
**Patient Perspective**	12	The patient should share their perspective in one to two paragraphs on the treatment(s) they received	N/A
**Informed Consent**	13	Did the patient give informed consent? Please provide if requested	**Yes** ☑ **No** ☐
